# Multifactor dimensionality reduction reveals a three-locus epistatic interaction associated with susceptibility to pulmonary tuberculosis

**DOI:** 10.1186/1756-0381-6-4

**Published:** 2013-02-18

**Authors:** Ryan L Collins, Ting Hu, Christian Wejse, Giorgio Sirugo, Scott M Williams, Jason H Moore

**Affiliations:** 1 Institute for Quantitative Biomedical Sciences, Dartmouth College, Hanover NH 03755, USA; 2 Department of Genetics, Geisel School of Medicine, Dartmouth College, Hanover NH 03755, USA; 3 Bandim Health Project, Danish Epidemiology Science Centre and Statens Serum Institute, Bissau, Guinea-Bissau and Center for Global Health, School of Public Health, Aarhus University, Skejby, Denmark; 4 Ospedale San Pietro FBF, Research Center, Rome, Italy

**Keywords:** Epistasis, Gene-gene interactions, Machine learning, Pulmonary tuberculosis

## Abstract

**Background:**

Identifying high-order genetics associations with non-additive (i.e. epistatic) effects in population-based studies of common human diseases is a computational challenge. Multifactor dimensionality reduction (MDR) is a machine learning method that was designed specifically for this problem. The goal of the present study was to apply MDR to mining high-order epistatic interactions in a population-based genetic study of tuberculosis (TB).

**Results:**

The study used a previously published data set consisting of 19 candidate single-nucleotide polymorphisms (SNPs) in 321 pulmonary TB cases and 347 healthy controls from Guniea-Bissau in Africa. The ReliefF algorithm was applied first to generate a smaller set of the five most informative SNPs. MDR with 10-fold cross-validation was then applied to look at all possible combinations of two, three, four and five SNPs. The MDR model with the best testing accuracy (TA) consisted of SNPs rs2305619, rs187084, and rs11465421 (TA = 0.588) in PTX3, TLR9 and DC-Sign, respectively. A general 1000-fold permutation test of the null hypothesis of no association confirmed the statistical significance of the model (p = 0.008). An additional 1000-fold permutation test designed specifically to test the linear null hypothesis that the association effects are only additive confirmed the presence of non-additive (i.e. nonlinear) or epistatic effects (p = 0.013). An independent information-gain measure corroborated these results with a third-order epistatic interaction that was stronger than any lower-order associations.

**Conclusions:**

We have identified statistically significant evidence for a three-way epistatic interaction that is associated with susceptibility to TB. This interaction is stronger than any previously described one-way or two-way associations. This study highlights the importance of using machine learning methods that are designed to embrace, rather than ignore, the complexity of common diseases such as TB. We recommend future studies of the genetics of TB take into account the possibility that high-order epistatic interactions might play an important role in disease susceptibility.

## Findings

### Introduction

Understanding the genetic architecture of common human diseases such as tuberculosis (TB) remains one of the greatest challenges in biomedical research. The goal of the present study was to approach the genetic analysis of TB susceptibility with the assumption that the underlying genetic architecture is complex.

Specifically, we used a machine learning method called multifactor dimensionality reduction (MDR) that was designed specifically for detecting and characterizing non-additive gene-gene interactions (i.e. epistasis) 1. Only a handful of studies have explored the role of epistasis in determining TB susceptibility. For example, de Wit et al. 2found statistically significant evidence for epistasis between several different pairs of single-nucleotide polymorphisms (SNPs) in a study of South Africans. Another study of West Africans found significant evidence of pairwise epistasis 3. We have extended these studies by specifically testing higher-order models of gene-gene interactions using machine learning methods.

The MDR method was designed as a machine learning alternative to parametric statistical methods such as logistic regression 1. The goal of MDR is to recode SNP data using constructive induction to make non-additive interactions easier to detect 4. Simulation studies have demonstrated that MDR has good power to detect non-additive epistatic interactions in the absence of detectable main effects 5,6. MDR has been applied to numerous genetic studies of common diseases including TB 3.

As with any machine learning method, there is always the concern of overfitting that can lead to false-positives. To avoid this problem here, we implemented MDR in a cross-validation framework that assesses the predictive ability of the models 7. We also performed rigorous permutation testing methods to assess how often MDR models as good as the ones we observed in the real data were found under the null hypothesis 8. As an additional measure, we implemented a ReliefF filter to reduce the total number of SNPs and thus SNP combinations evaluated by MDR 9. This greatly reduces the total number of tests performed.

### Methods

The data set used in this study was originally analyzed by Olessen et al. 3. These data include 321 pulmonary TB cases and 347 healthy controls genotyped at The Bandim Health Project in Guinea Bissau 3. Each individual was genotyped for 19 single-nucleotide polymorphisms (SNPs) from immunological candidate genes VDR, DC-SIGN, PTX3, TLR2, TLR4, and TLR9. Missing data were imputed using a frequency-based imputation. Additional details about the choice of genes and the overall study are provided by Olessen et al. 3.

We first applied the ReliefF algorithm to filter the 19 SNPs to a total of five. Here, we used 100 nearest neighbors. The goal for the ReliefF analysis was to retain only those SNPs that provide the greatest signal. This reduces the total number of models that need to be explored.

We then applied MDR to five filtered SNPs. We combinatorially evaluated all two-way to five-way models. Balanced accuracy in the context of 10-fold cross-validation was used to assess model quality. An overall best model was selected that had the maximum accuracy in the testing data (i.e. testing accuracy or TA). We also recorded the cross-validation consistency or CVC. This provides a summary of the number of cross-validation intervals in which a particular model was found. Higher numbers indicate more robust results. Statistical significance was assessed using 1000-fold permutation testing. Here, the data are randomized 1000 times to create 1000 datasets consistent with the null hypothesis. The complete MDR analysis is repeated in each permutated dataset and a best model selected just as in the real data. This procedure generates an empirical estimate of the null distribution of testing accuracies and corrects for multiple testing because the same number of models are evaluated in all permutated and real data. We performed two tests. First, we tested the general null hypothesis of no association by randomizing case–control labels. Second, we tested the linear null hypothesis that the only genetic effects are additive according the genotype randomization methods of Greene et al. 8. Rejection of both null hypotheses is evidence for non-additive epistasis. We considered all result significant at the alpha = 0.05 level.

In addition to the MDR analysis, we performed an independent assessment of non-additivity using entropy-based measures of information gain 4. Specifically, we used a new measure of three-way epistasis that adjusts for lower-order effects 10. This approach was used to confirm high-order non-additive interactions.

### Results

**Figure 1:**
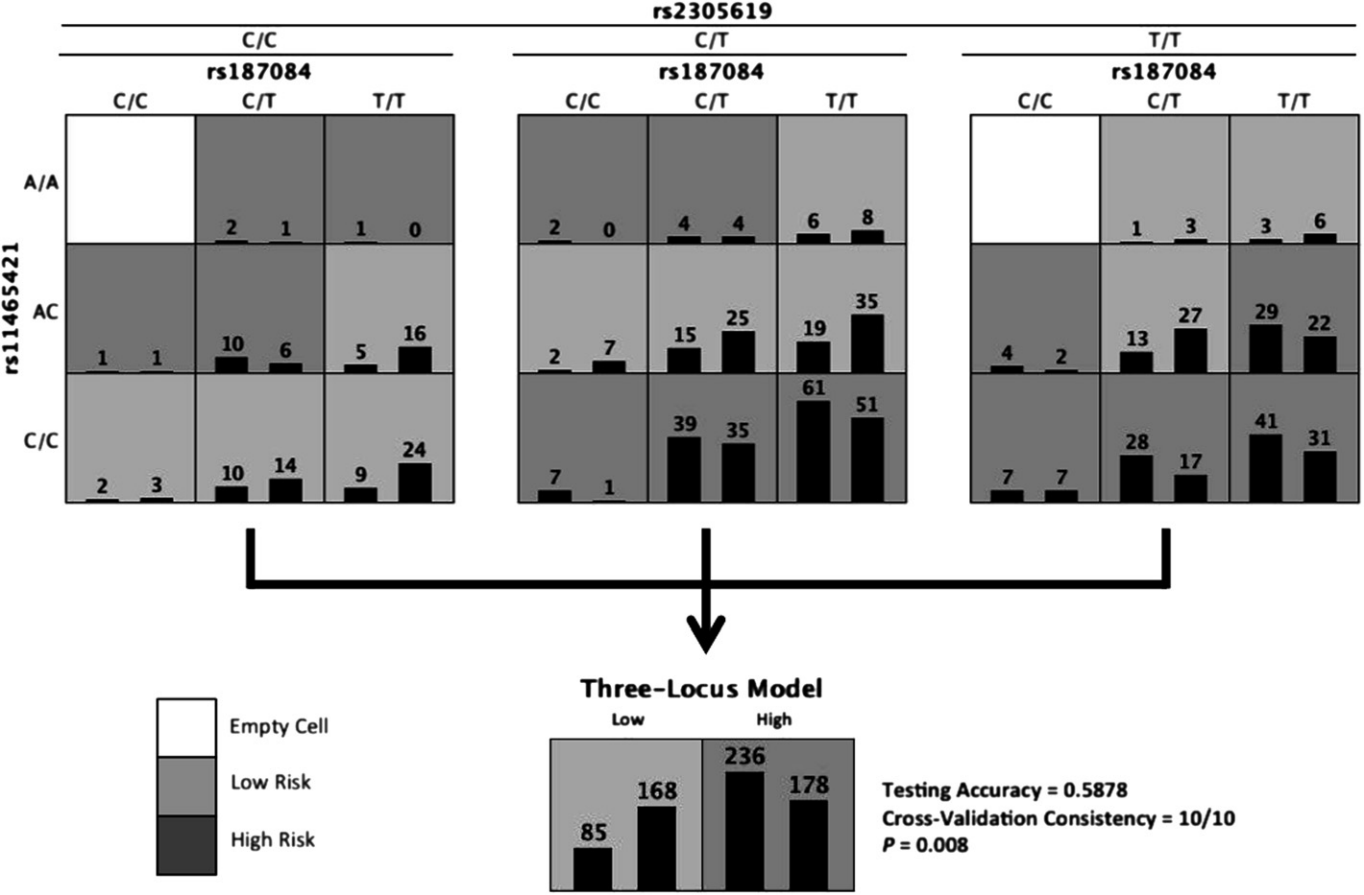
**Distribution of cases (left bars) and controls (right bars) for each genotype combination from the three single nucleotide polymorphisms identified in the overall best model by multifactor dimensionality reduction (MDR) analysis.** High-risk genotypes are shaded *dark grey* and low-risk genotypes are shaded *light grey*. The new variable constructed by MDR is shown on the *right*.

ReliefF filtering returned the following five SNPs (corresponding genes shown in parentheses): rs187084 (TLR9), rs4986790 (TLR4), rs11465421 (DC-SIGN), rs2305619 (PTX3), rs1840680 (PTX3), and rs2287886 (DC-SIGN). A summary of the MDR results for these five SNPs is shown in Table 1. None of the SNPs were found to have statistically significant main effects after correction for multiple testing. Additionally, no statistically significant pairwise models were reported. The overall best model consisted of SNPs rs2305619, rs187084, and rs1145421. These three SNPs had a training accuracy of 0.6115 and a testing accuracy of 0.5878. The cross-validation consistency of this model was 10/10. The distribution of cases and controls for each of the three-locus genotype combinations in the best MDR model can be seen in Figure 1.

Permutation testing confirmed the statistical significance of the model suggesting it is unlikely to see a mode this good in null data (p = 0.008). Additional permutation testing revealed that the non-additive effects in the model were also statistically significant (p = 0.013). Taken together, these results suggest a role for high-order non-additive epistatic effects.

**Figure 2:**
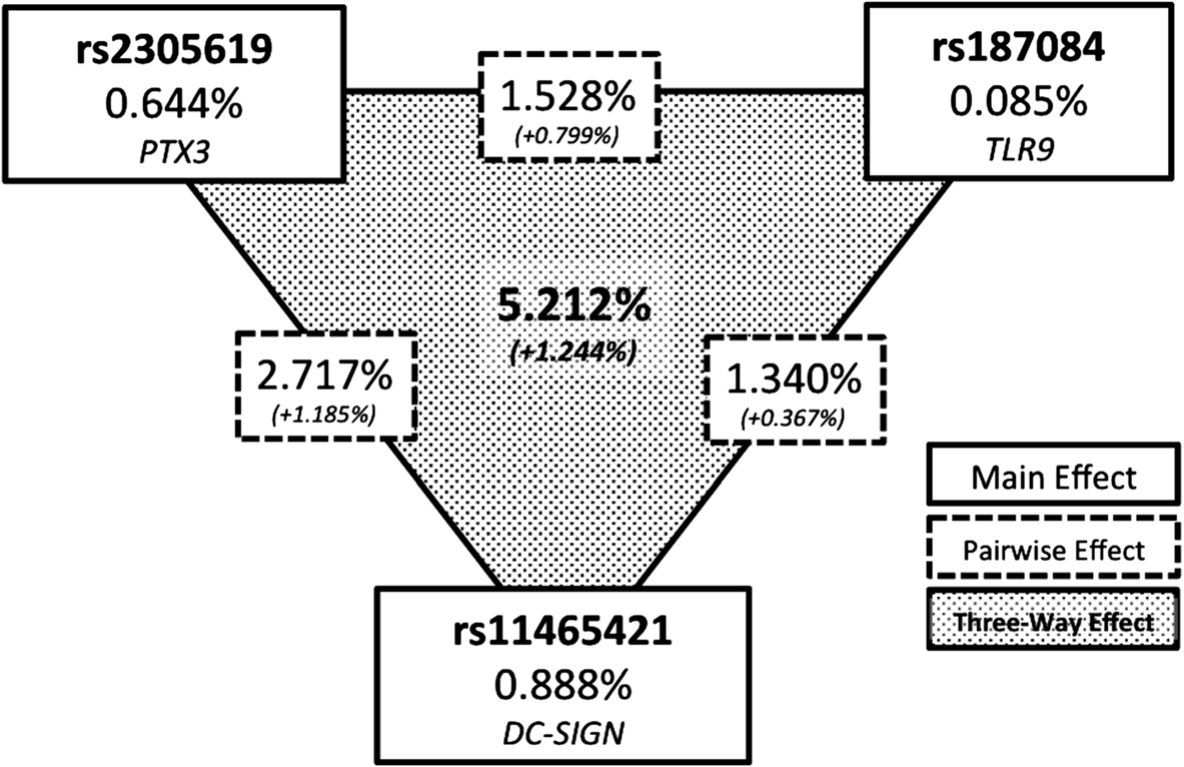
**Summary of information gain by main effects (solid borders), pairwise effects (dashed borders), and the three-way effect (shaded) of single nucleotide polymorphisms (SNPs) found in the overall best model of multifactor dimensionality reduction.** Relative synergy or redundancy of each model is indicated below the principal effect in italics. The gene associated with each SNP is indicated below each main effect.

Figure 2 summarizes the results of the entropy-based information gain analysis. We found that the three-way epistatic interaction was stronger than any lower-order effects. This confirms the results we observed with MDR.

### Discussion

Few studies consider the role of epistasis in disease susceptibility. Even fewer consider the possibility that multiple genetic variants might have synergistic effects beyond main effects or pairwise effects. We have demonstrated how the ReliefF and MDR machine learning algorithms can be employed in conjunction with cross-validation and permutation testing to move beyond the detection of low-order genetic effects. We have applied these approaches to the genetic analysis of TB susceptibility and have demonstrated a statistically significant three-way epistatic interaction exhibiting non-additivity that is not predicted by the one-way and two-way effects. These results were confirmed using an independent analysis approach based on information theory. An important question is whether this three-locus epistatic effect has biological and clinical implications. The biological connection is not difficult given these genes were pre-selected as good immunological candidates for TB 3. Whether the genetic effects specified in the model are functional will need to be determined by experimental methods. Application of these methods and other machine learning approaches will be important for unraveling the genetic complexity of TB.

## Availability

All methods are freely available as open-source software from the authors. More information can be found at http://epistasis.org.

## Abbreviations

MDR: Multifactor dimensionality reduction; SNP: Single-nucleotide polymorphism; TB: Tuberculosis

## Competing interests

The authors declare no competing interests.

## Authors’ contributions

RC performed the data analysis and drafted the manuscript. TH imputed missing data, performed information gain measurements and critiqued manuscript drafting. CW, GS and SW provided and summarized the data, assisted with results interpretation and assisted with the writing. JM conceived of the study, provided administrative direction, assisted with results interpretation, assisted with the writing, and secured financial support for the study. All authors read and approved the final manuscript.

## References

[B1] Ritchie MD Hahn LW Roodi N Bailey LR Dupont WD Parl FF Moore JH Multifactor-dimensionality reduction reveals high-order interactions among estrogen-metabolism genes in sporadic breast cancer Am J Hum Genetics 2001 69 138 147 10.1086/321276 11404819 PMC1226028

[B2] De Wit E van der Merwe L van Helden PD Hoal EG Gene-gene interaction between tuberculosis candidate genes in a South African population Mamm Genome 2011 22 1–2 100 110 20799037 10.1007/s00335-010-9280-8

[B3] Olesen R Wejse C Velez DR Bisseye C Sodemann M Aaby P Rabna P Worwui A Chapman H Diatta M Adegbola RA Hill PC Østergaard L Williams SM Sirugo G DC-SIGN (CD209), pentraxin 3 and vitamin D receptor gene variants associate with pulmonary tuberculosis risk in West Africans Genes Immun 2007 8 suppl 6 456 467 17611589 10.1038/sj.gene.6364410

[B4] Moore JH Gilbert JC Tsai C Chiang F Holden T Barney N White BC A flexible computational framework for detecting, characterizing, and interpreting statistical patterns of epistasis in genetic studies of human disease susceptibility J Theor Biol 2006 241 252 261 10.1016/j.jtbi.2005.11.036 16457852

[B5] Ritchie MD Hahn LW Moore JH Power of multifactor dimensionality reduction for detecting gene-gene interactions in the presence of genotyping error, missing data, phenocopy, and genetic heterogeneity Genet Epidemiol 2003 24 2 150 157 10.1002/gepi.10218 12548676

[B6] Velez DR White BC Motsinger AA Bush WS Ritchie MD Williams SM Moore JH A balanced accuracy function for epistasis modeling in imbalanced datasets using multifactor dimensionality reduction Genet Epidemiol 2007 31 4 306 315 10.1002/gepi.20211 17323372

[B7] Coffey CS Hebert PR Ritchie MD Krumholz HM Gaziano JM Ridker PM Brown NJ Vaughan DE Moore JH An application of conditional logistic regression and multifactor dimensionality reduction for detecting gene-gene interactions on risk of myocardial infarction: the importance of model validation BMC Bioinformatics 2004 5 49 10.1186/1471-2105-5-49 15119966 PMC419697

[B8] Greene CS Himmelstein DS Nelson HH Kelsey KT Williams SM Andrew AS Karagas MR Moore JH Enabling personal genomics with an explicit test of epistasis Pac Symp Biocomput 2010 327 336 19908385 10.1142/9789814295291_0035 PMC2916690

[B9] Greene CS Penrod NM Kiralis J Moore JH Spatially uniform ReliefF (SURF) for computationally-efficient filtering of gene-gene interactions Biodata Min 2009 2 5 13 10.1186/1756-0381-2-5 19772641 PMC2761303

[B10] Hu T Chen Y Kiralis JW Collins RL Wejse C Sirugo G Williams SM Moore JH An information-gain approach to detecting three-way epistatic interactions in genetic association studies J Am Med Inform Assoc 2013 10.1136/amiajnl-2012-001525 PMC3721169 23396514

